# AGE-SPECIFIC DETERMINANTS OF PHYSICAL ACTIVITY ENGAGEMENT IN BREAST CANCER SURVIVORS: A MULTIGROUP ANALYSIS

**DOI:** 10.1093/geroni/igae098.3784

**Published:** 2024-12-31

**Authors:** Mi Sook Jung, Xirong Cui, Kyeongin Cha, Ahrim Lee, Eunyoung Park, Hyun-E Yeom

**Affiliations:** Chungnam National University, Daejeon, Republic of Korea; Chungnam National University, Daejeon, Republic of Korea; Chungnam National University, Daejeon, Republic of Korea; Chungnam National University, Daejeon, Republic of Korea; Chungnam National University, Daejeon, Republic of Korea; Chungnam National University, Daejeon, Republic of Korea

## Abstract

Physical activity is highly beneficial for breast cancer survivors. However, many survivors often have insufficient levels of physical activity due to fatigue, psycho-social barriers, limited knowledge, and low self-efficacy. These barriers vary in type and severity due to age-related factors, potentially leading to differences in physical activity levels and health-related quality of life (HRQOL). This study drew upon the Individual and Family Self-Management Theory to explore the interrelationships between contextual and process factors and proximal outcomes (physical activity, HRQOL). Community-dwelling 450 breast cancer survivors were recruited to assess cancer stigma, health literacy, social support, exercise self-efficacy, behavioral regulation, physical activity engagement, and HRQOL. Structural equation modeling and multigroup analysis with the moderating effect of age were performed. Greater stigmatization of cancer, lower social support, and worse behavioral regulation were directly associated with lower physical activity engagement while health literacy, behavioral regulation, and physical activity engagement had direct effects on HRQOL. Behavioral regulation mediated the association between contextual factors (stigma, health literacy) and physical activity. After confirming full measurement invariance between the groups, significant differences in factor loadings were found in specific pathways from contextual (stigma) and process factors (behavioral regulation, social support) to physical activity, highlighting age-related differences in how these factors interact. The findings underscore how complex patterns of contextual and process correlates influence physical activity in older and middle-aged women with breast cancer, respectively. The age-specific uniqueness should be considered when developing targeted strategies to promote active living and achieve optimal health in later life.

